# 

**Published:** 1882-03

**Authors:** 


### Pamphlets.

Transactions of the twenty-eighth annual meeting of the Medical Society of the State of North Carolina, held at Ashville, May 31, 1881. Dr. Thomas F. Wood, Wilmington, President; Dr. L. Julien Picot, Littleton, Secretary.

The fourth annual report of the Presbyterian Eye and Ear Charity Hospital, Baltimore. Julian J. Chisolm, M.D., surgeon in charge.

Soluble Compressed Pellets, a new form of remedies for hypodermic use, and applicable to ophthalmia and general medication. By H. Augustus Wilson, M.D. Reprint from the Transactions of the American Medical Association, 1881.

Chronic Club Foot successfully treated without tenotomy, by continuous extension and stretching. By James S. Green, M.D. Reprint from the *New York Medical Journal and Obstetrical Review.*, November, 1881.

Insanity in its Relations to the Medical Profession and Lunatic Hospitals. By Nathan Allen, M.D. Read at the annual meeting of the American Association for the Protection of the Insane in New York.

Removal of a Cyst of the Pancreas weighing twenty and one-half pounds. By Nathan Bozeman, M.D. Proceedings of the New York Pathological Society.

